# Congenital Pulmonary Airway Malformation with Pulmonary Arteriovenous Malformation in Adulthood: A Case Report

**DOI:** 10.70352/scrj.cr.25-0176

**Published:** 2025-06-03

**Authors:** Takehiro Suzuki, Naohiro Kobayashi, Yohei Yatagai, Shinsuke Kitazawa, Hideo Ichimura, Yukio Sato

**Affiliations:** 1 Department of General Thoracic Surgery, Tsukuba University Hospital, Tsukuba, Ibaraki, Japan; 2 Department of Pulmonary Medicine, Tsukuba University Hospital, Tsukuba, Ibaraki, Japan

**Keywords:** congenital pulmonary airway malformation, pulmonary arteriovenous malformation, aspergillosis

## Abstract

**INTRODUCTION:**

Congenital pulmonary airway malformation (CPAM) is a congenital condition rarely detected in adults because most cases of CPAM are found through prenatal testing or through testing for recurrent pneumonia or lung abscesses in childhood. Pulmonary arteriovenous malformation (PAVM) is an abnormal vascular connection between the pulmonary arteries and veins, which is often related to hereditary hemorrhagic telangiectasia, but can also be induced by infections, trauma, or thoracic surgery. Herein, we report an adult case of coexisting CPAM and PAVM.

**CASE PRESENTATION:**

The patient was a 26-year-old woman. A medical checkup chest X-ray showed abnormalities. The patient had no past medical history, including of bleeding tendency or repeated pneumonia, and no familial history of CPAM and PAVM. A chest CT revealed multiple lung cysts (maximum diameter of 40 mm) in the left lower lobe of the lung, and congenital pulmonary cysts were suspected. The chest CT also showed two PAVMs (vessel diameters of 6 mm and 4 mm) in the same left lower lobe. Serum tests were positive for *Aspergillus*-specific antibodies and β-D-glucan, and pulmonary aspergillosis was diagnosed. An antifungal agent (itraconazole) was administered. However, consolidations had developed 9 months after, and the antifungal agent was changed to voriconazole. Then, the consolidations diminished slightly but nevertheless remained, and one of the PAVMs increased in diameter from 6 mm to 10 mm. A left lower lobectomy under thoracoscopy was performed owing to the uncontrolled infection and the risk of complications with PAVMs. The pathological diagnosis of the pulmonary cysts was CPAM type 1. The patient had no symptoms or complications after the surgery.

**CONCLUSIONS:**

Cases of CPAM with PAVM are rare, especially in adults. CPAM often leads to pulmonary infection, and the pulmonary infection is known to be one of the causes of PAVM. In our case, *Aspergillus* might have infected the pulmonary cysts and affected the enlargement of the vascular diameter of PAVM. If CPAM and PAVM are present simultaneously, surgical treatment should be considered to prevent complications associated with CPAM and PAVM.

## Abbreviations

CPAM
: congenital pulmonary airway malformation
S9
: lateral basal segment
S10
: dorsobasal segment
PAVM
: pulmonary arteriovenous malformation

## INTRODUCTION

Congenital pulmonary airway malformation (CPAM) is a congenital condition that leads to multiple cysts caused by adenomatous hyperplasia of the bronchiolar epithelium. CPAMs are rarely found in adults.^1–3)^ Approximately 80% of CPAMs are detected prenatally or diagnosed during the neonatal period, when patients initially present with dyspnea or cyanosis. Most CPAMs not detected in the neonatal period are found in infants or school-aged children with recurrent pneumonia and/or lung abscesses. Although CPAMs are often accompanied by infections,^2)^ few present with vascular anomalies. Here, we report a rare case of CPAM type 1 and pulmonary arteriovenous malformations (PAVMs), which was complicated by pulmonary aspergilloma.

## CASE PRESENTATION

**Fig. 1:**
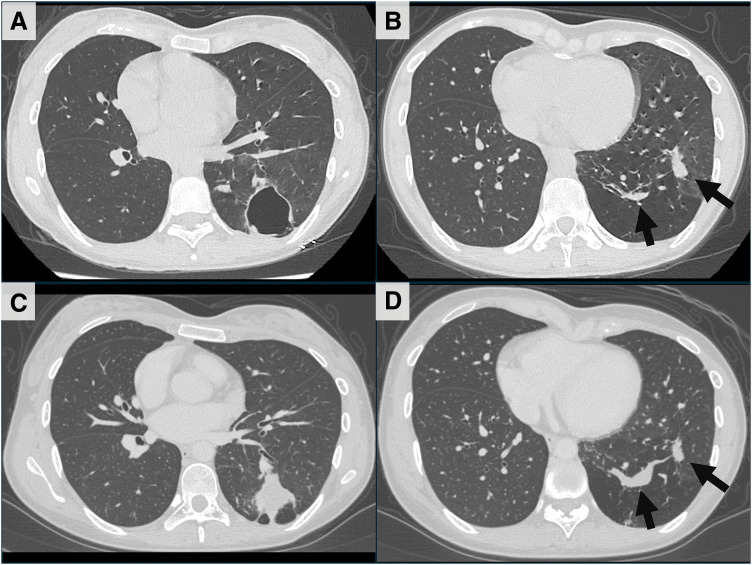
CT images at the time of the initial visit (**A**, **B**) and preoperatively (**C**, **D**). (**A**) Lung cyst with a maximum diameter of 40 mm in the left lower lobe. (**B**) PAVMs (arrows). (**C**) Lung cyst with fluid accumulation. (**D**) PAVMs with dilated diameter (arrows).

**Fig. 2:**
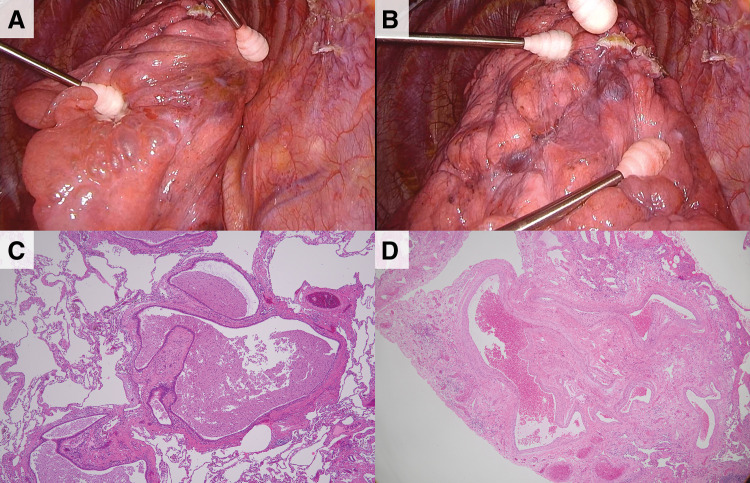
Thoracoscopic findings (**A**, **B**) and microscopic findings (**C**, **D**). (**A**) One yellow lung cyst and several clear lung cysts were seen just below the visceral pleura. (**B**) PAVMs were found near pleural adhesions. (**C**) The lung cysts were lined with bronchial epithelium and contained necrotic tissue with numerous macrophages. (**D**) PAVM findings showed an accumulation of vessels of various sizes.

The patient was a 26-year-old woman. She initially visited our hospital because of an abnormal shadow found on a chest X-ray at a medical checkup 2 years before. She did not have any symptoms or past medical history including of bleeding tendency or repeated pneumonia. No one in her family had CPAM or PAVMs. A chest X-ray showed cysts and consolidations in the left lower lung field. A chest CT revealed multiple lung cysts (maximum diameter of 40 mm, **Fig. 1A**) and several nodules in the left lower lobe of the lung. The CT also showed 2 PAVMs (**Fig. 1B**). One was in the dorsobasal segment (S10) and A10 flowed into V10 with 6 mm of vessel diameter, and the other was in the lateral basal segment (S9) and A9 flowed into V9 with 4 mm of vessel diameter. As serology tests were positive for *Aspergillus*-specific antibodies and β-D-glucan was 18 pg/mL (reference value <10.9 pg/mL), aspergillosis was clinically diagnosed and treatment with itraconazole initiated, although inflammatory findings were not observed. However, consolidations had developed 9 months later. The antifungal agent was changed to voriconazole. Eighteen months after her first visit, a chest CT showed that consolidations had diminished slightly, but remained (**Fig. 1C**). The PAVM in S10 was enlarged to 10 mm in diameter (**Fig. 1D**). The cysts were localized to the left lower lobe only, and the aspergillosis was smoldering despite the continued administration of the antifungal medication. Considering these conditions, we performed a left lower lobectomy under thoracoscopy. The surgical findings showed strong adhesions between the left lower lobe and the chest wall, dilated and tortuous bronchial arteries, PAVMs on the visceral pleura, and yellowish nodules in the left lower lobe (**Fig. 2A**, **2B**). The patient was discharged 7 days after surgery without any complications. Macroscopically, multiple cysts were present in the left lower lobe with a maximum diameter of 30 mm. Microscopically, the cysts were lined with bronchial epithelium and contained necrotic tissue with numerous macrophages. Smooth muscle bundles were present around the cysts, but no skeletal muscle or cartilage tissue was identified (**Fig. 2C**). The pathological diagnosis was CPAM type 1. No pathogens stained with Gram, PAS, Grocott, or Ziehl-Neelsen were detected. Additionally, the findings of PAVM were observed in the basal segment, which is characterized by accumulations of vessels of various sizes without associated bronchioles or interlobar septa (**Fig. 2D**). The patient had no symptoms or complications 6 months after surgery. Voriconazole was medicated until 3 months postoperatively.

## DISCUSSION

CPAM type 1 and PAVMs coexisted in our case. CPAM, previously known as congenital cystic adenomatoid malformation, was classified into types I to III by Stocker in 1977 and then reclassified into types 0 to 4 in 2002.^1,4)^ The classification is based on the area of the cysts, the cyst size, and the cyst appearance. Type 1 is a lesion of the bronchial/bronchiolar area, composed of one to several cysts of relatively large size (1–10 cm) and mimicking the distal bronchial tree and proximal acinus with its bronchi-like and proximal bronchiole-like structures.^4)^ Type 1 is the most common CPAM (approximately 65% of all cases). CPAM type 1 cysts are limited to 1 lobe in about 95% of cases. A relationship of CPAM type 1 to lung cancer has been suggested.^3,4)^

PAVMs are abnormal vascular connections directly between the pulmonary arteries and veins. Their prevalence is as low as 2 to 38 per 100,000 population.^5–8)^ Seventy to ninety percent of PAVMs are associated with hereditary hemorrhagic telangiectasia.^6,9)^ In non-hereditary hemorrhagic telangiectasia patients, the underlying causes have been reported to be previous thoracic surgery, congenital heart disease, history of chest trauma, and history of pulmonary infection or abscess.^8,10)^ PAVMs are mostly asymptomatic but occasionally present with symptoms related to right-to-left shunt (hemoptysis, dyspnea, and cyanosis) and systemic embolization (cerebral abscess and stroke). Treatment with a transcatheter or with surgery is recommended for symptomatic patients and for asymptomatic patients when the size is larger than 20 mm, or when the diameter of the inflow artery is larger than 3 mm because of the possibility of severe complications.^11)^

To the best of our knowledge, this is the first case report of CPAM with PAVMs. One possible reason for the simultaneous presence of CPAM and PAVMs is that the CPAM was congenital and the PAVMs were acquired. Pulmonary infection is one cause of PAVMs, and Albitar et al. reported that 7.8% of non-hereditary PAVMs had a history of pulmonary infection as a possible cause.^8)^ In our case, pulmonary aspergillosis seen in the lung with CPAM might have been a cause of the PAVMs. The finding that the size of the PAVMs increased over a relatively short period of time (18 months) supports the possibility that the PAVMs were acquired rather than congenital. Another possible reason is that both the CPAM and the PAVMs were congenital. The fact that most PAVMs are congenital^5)^ supports this possibility. One of the speculations is that CPAM caused the formation of abnormal lung tissue and the pulmonary arteries connected directly to the pulmonary veins without alveolar capillaries during the fetal period. However, we considered this likelihood relatively low because the patient had no family history of CPAM or PAVMs. The lack of reports in the literature on the coexistence of CPAM and PAVMs may also indicate that the probability of both CPAM and PAVMs occurring during the embryonic period is quite low.

In our case, left lower lobectomy was chosen as the treatment because the CPAMs were located only in the left lower lobe, the CPAMs were complicated by aspergillosis that was uncontrolled with antifungal agents, and PAVMs were also present and dilated. The relationship of CPAM type 1 to lung cancer was a complementary reason for surgery in our case. Fortunately, complications related to PAVMs, such as embolization or rupture, did not develop; nevertheless, surgery should have been performed earlier, given that the PAVMs were enlarged by *Aspergillus* infection. The surgery could be completed without any troubles under thoracoscopy, although inflammatory changes, such as tight pleural adhesions and tortuous bronchial arteries, were seen.

## CONCLUSIONS

We have reported here an adult case of CPAM with PAVMs. It was possible that *Aspergillus* infected the CPAMs and that the infection caused dilatation of the diameter of the PAVMs. When CPAM and PAVM are present simultaneously, surgical treatment should be considered to prevent complications associated with CPAM and PAVM.

## ACKNOWLEDGMENTS

We appreciate Ms. Flaminia Miyamasu, Medical English Communications Center, University of Tsukuba, for revision of this manuscript.

## DECLARATIONS

### Funding

The authors received no specific funding for this work.

### Authors’ contributions

T.S. and N.K. designed and drafted the manuscript.

S.K., H.I., Y.S., and Y.Y. reviewed and revised the manuscript.

All the authors have read and approved the final manuscript.

### Availability of data and materials

Not applicable.

### Ethics approval and consent to participate

This work does not require ethical considerations or approval. Informed consent to participate in this study was obtained from the patient.

### Consent for publication

Informed consent was obtained from the patient for the publication of this case report and any accompanying images.

### Competing interests

The authors declare that they have no competing interests and availability of data and materials.
